# Wearable sensors for monitoring the internal and external workload of the athlete

**DOI:** 10.1038/s41746-019-0149-2

**Published:** 2019-07-29

**Authors:** Dhruv R. Seshadri, Ryan T. Li, James E. Voos, James R. Rowbottom, Celeste M. Alfes, Christian A. Zorman, Colin K. Drummond

**Affiliations:** 1 Department of Biomedical Engineering, Case Western Reserve University, 10900 Euclid Avenue, Cleveland, OH 44106 USA; 2 Department of Orthopaedic Surgery, University Hospitals Cleveland Medical Center, Cleveland, OH 44106 USA; 3 University Hospitals Sports Medicine Institute, Cleveland, OH 44106 USA; 4 Department of Cardiothoracic Anesthesiology, The Cleveland Clinic, 9500 Euclid Avenue, Cleveland, OH 44195 USA; 5 Frances Payne Bolton School of Nursing, Case Western Reserve University, 9501 Euclid Avenue, Cleveland, OH 44106 USA; 6 Department of Electrical Engineering and Computer Science, Case Western Reserve University, 10900 Euclid Avenue, Cleveland, OH 44106 USA

**Keywords:** Translational research, Data acquisition

## Abstract

The convergence of semiconductor technology, physiology, and predictive health analytics from wearable devices has advanced its clinical and translational utility for sports. The detection and subsequent application of metrics pertinent to and indicative of the physical performance, physiological status, biochemical composition, and mental alertness of the athlete has been shown to reduce the risk of injuries and improve performance and has enabled the development of athlete-centered protocols and treatment plans by team physicians and trainers. Our discussions in this review include commercially available devices, as well as those described in scientific literature to provide an understanding of wearable sensors for sports medicine. The primary objective of this paper is to provide a comprehensive review of the applications of wearable technology for assessing the biomechanical and physiological parameters of the athlete. A secondary objective of this paper is to identify collaborative research opportunities among academic research groups, sports medicine health clinics, and sports team performance programs to further the utility of this technology to assist in the return-to-play for athletes across various sporting domains. A companion paper discusses the use of wearables to monitor the biochemical profile and mental acuity of the athlete.

## Introduction

**Fig. 1:**
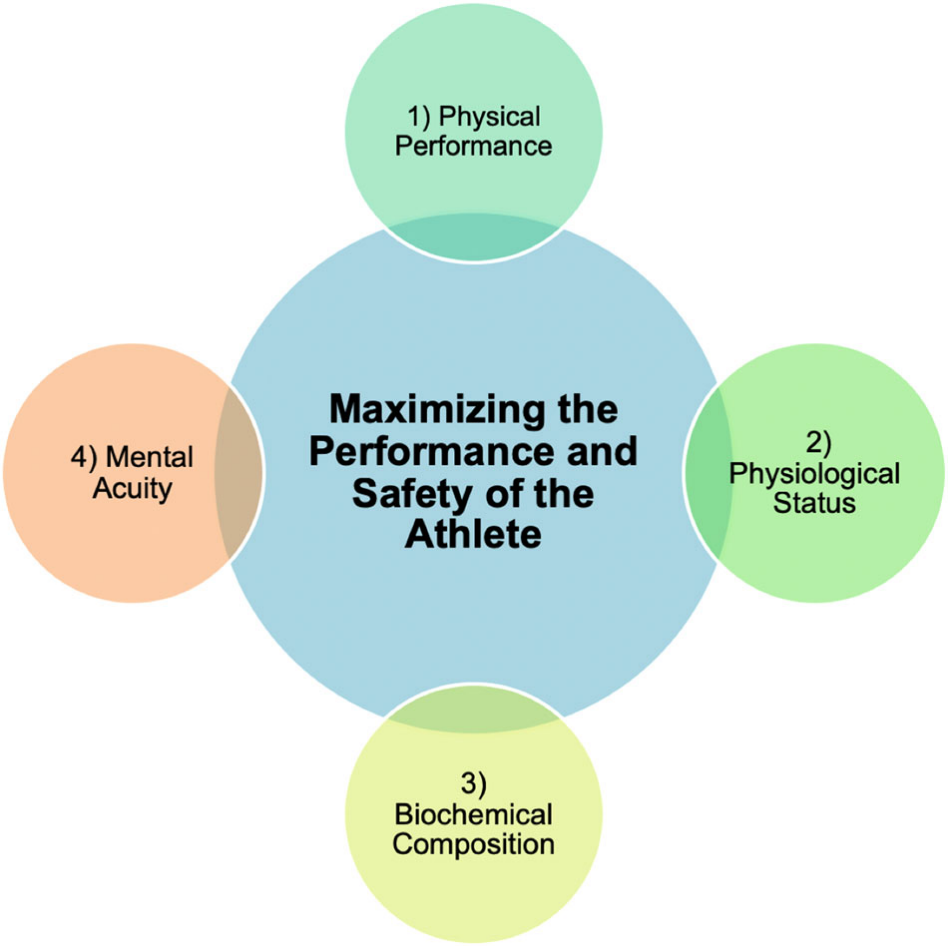
Four areas of focus as it relates to assessing human performance. The central theme of this review is the use of wearable sensors to maximize the performance and safety of the athlete. This involves the detection and measurement of the internal and external workload of the athlete which are based on the athlete’s physical performance, physiological status, biochemical composition, and mental acuity

Technological advancements have enabled athletes, coaches, and physicians to track functional movements, workload, biomechanical and bio-vital markers utilizing wearable sensors to maximize performance and minimize the potential for injury.^1–3^ Wearable monitoring systems can provide continuous physiological data thus permitting the development of accurate treatment plans and player-specific training programs to potentially mitigate and alleviate injuries.^4^ Herein, we define a wearable device as a sensor or sensor suite unencumbered by wires for the continuous and non-invasive detection of biosignals, analytes, or biomechanical and impact forces for monitoring human health and performance. Over the past two decades, the wearables field has moved from a *device* to a *systems* viewpoint, where the system combines the device with analytics. While previous literature has reviewed specific technical domains of the wearables field, such as sensors,^5–8^ materials,^9–12^ and soft interfaces^13–15^ or focused on the fabrication and application of such devices to address a specific medical condition, such as atrial fibrillation,^16–18^ cystic fibrosis,^19–21^ or diabetes,^22–27^ there remains an unmet medical need to assess, develop, and validate this technology specifically for sports medicine. Given the heightened attention to athlete safety and performance, this review evaluates the translational utility of wearable devices to detect key metrics pertinent to human performance assessment (Fig. 1).

The organization of this review is structured around discussing the value wearable sensors provide in sports to monitor player activity levels and mitigate injury. We progress through this review by discussing the utility of wearable sensors in two domains crucial to human performance ranging from an athlete’s physical performance and physiological status. Our goal in each of these areas is centered around reviewing both scientific literature and current commercially available devices to provide a comprehensive view of wearables for sports medicine (Tables 1–5). This review is specifically targeted towards those whose interests lie in the application and translation of wearable sensors for assessing human performance.

**Table 1:** Examples of wearable technology companies with products applicable towards assessing the position and motion of the athlete

Company	Sampling of products	Product type	Product functionality	Headquarters
Adidas	miCoach Fit Smart, miCoach Smart Run	Watch	Heart rate, GPS, distance	Herzogenaurach, Germany
Apple	Apple Watch	Watch	Heart rate, distance, email, ECG, text messages, phone	Cupertino, CA
BioSensive Technologies Inc.	Joule	Earrings	Heart rate, calories burned, steps taken, overall activity level	Ontario, Canada
Catapult	OptimEye S5, Vector	Device unit	Movement, Turn rates, orientation, heart rate. Device placed below the neck (tucked in shoulder pads)	Melbourne, Australia
Fitbit	Flex, One, Alta	Watch	Steps walked, distance, heart rate, sleep quality, pedometer, calories burned	San Francisco, CA
Garmin	Vivoactive, Vivosmart, Vivofit	Watch	Pedometer, sleep quality, heart rate, distance	Schaffhausen, Switzerland
Jabra	Sports Pulse Wireless Headphone	Headphone	Accelerometer and heart rate monitoring	Ballerup, Denmark
Jawbone	Up	Band	Pedometer, distance, heart rate, sleep quality, calories	San Francisco, CA
Karacus	Polaris, Zeta, Proxima	Watch	Movement, phone, email	Chapel Hill, NC
Kitman Labs	Capture	Sensor mounted on computer	Biometric data via machine learning, GPS, and player tracking	Dublin, Ireland
Microsoft	Microsoft Band	Band	Heart rate, calories burned, sleep quality, email, text	Redmond, WA
Nike	Fuelband	Band	Pedometer, GPS	Washington County, Oregon
Polar	A360, Loop Crystal, Loop 2	Band	Heart rate, performance tracker	Kempele, Finland
Samsung	GearFit 2	Watch	GPS, sleep, heart rate, calories, pedometer	Seoul, South Korea
Sansible Technologies	LiveSkin	Textile electronics	Speed, impact, position	Edinburgh, United Kingdom
Starkey Hearing Technologies	Livio AI Hearing Aid	Hearing aid	Translates foreign languages, contains a pedometer, tracks physical activity (wellness score)	Eden Prairie, MN
Stifit	Stifit band	Band	Blood oxygen, body mass index, calories burned, distance, fatigue, heart rate, sleep	San Francisco, CA
TruSox	TruSox	Socks	Non-slip socks to generate greater speed and agility	Baltimore, MD
Under Armor	HTC Grip	Wristband	Heart rate, calories burned, distance traveled	Baltimore, MD
Vert	G-Vert, VERT Coach	Device unit	Measure G-force in movement, acceleration, kinetic energy, power	Fort Lauderdale, FL
Vibrado Technologies	Vibrado Technologies	Textile electronics	Accelerometer to measure shot angle, arm height, release point. Sleeve to be worn over forearm	Sunnyvale, CA
Zebra Technologies	Zebra Tracking Device	Device unit	RFID used to quantify movement and distance profiles. Device placed below the neck in shoulder pads or sewn into jersey	Lincolnshire, IL
Zephyr	BioHarness 3, HxM™ Smart, HxM™ BT	Textile Electronics	Heart rate, respiration, tri-axial accelerometer, heart rate, activity, posture, oxygen saturation levels	Annapolis, Maryland (Founded in New Zealand)

Data for this table was acquired from company websites and social media sites affiliated with each company

**Table 2:** Examples of wearable technology companies for impact monitoring

Company	Sampling of products	Product type	Product functionality	Headquarters
2ND Skull	Cap, Band	Garment	Polyurethane-based composite dissipates impact	Pittsburgh, PA
Athlete Intelligence	Vector Mouthguard, Shockbox® sensor	Mouth guard	Tracks linear and rotational accelerations of head impacts	Kirkland, WA
BrainScope	Ahead 300	Hand-held point of care device	Disposable electrode sensors to detect head injuries	Bethesda, MD
Force Impact Technologies	Fitguard™	Mouth guard	Embedded sensors relate collision intensity via color coded LED’s on the front of the mouth guard	Los Angeles, CA
				Tempe, AZ
Hiji	Hiji Band	Head band	Impact forces, intensity	Phoenix, AZ
Jolt	Jolt Sensor	Sensor	Impact forces, Concussion monitoring. Sensor clipped to garment	Boston, MA
Mamori	Mamori	Mouth guard	Inertial sensors measure impact forces on the head	Dublin, Ireland
Noggin Pro	Noggin, Noggin Pro	Skull caps	Gel capsules in skull cap dissipate forces from skull	Toronto, ON
Performance Sports Group	Q-Collar	Neck collar	Concussion prevention by applying pressure on the jugular vein	Cincinnati, OH
X2 Biosystems	X-Patch Pro	Flexible sensor	Tri-axial accelerometers to measure impact	Seattle, WA
	X2 Mouthguard			

Data for this table was acquired from company websites and social media sites affiliated with each company

**Table 3:** Examples of wearable technology companies for monitoring the biomechanical forces on the athlete

Company	Sampling of products	Product type	Product functionality	Headquarters
CricFlex	CricFlex	Sleeve	Measures arm angle and force during bowling	Islamabad, Pakistan
Heddoko	Heddoko	Smart Garment	Biomechanics of movement, deviation from benchmarks and movement standards, injury risk	Montreal, Canada
Motus Global	mThrow™, motusPro™	Sleeve	Accelerometer to measure joint angles, velocity, stress, strain	Massapequa, New York Ft. Lauderdale, FL
Protonics Technologies	Protonics T2	Device	Offsets left-right biomechanical imbalance to reduce muscle pain. Attached to left leg	Lincoln, NE

Data for this table was acquired from company websites and social media sites affiliated with each company

**Table 4:** Examples of wearable technology companies with products applicable towards monitoring heart rate and muscle oxygen saturation

Company	Sampling of products	Product type	Product functionality	Headquarters
1st Round Athletics	EnergyDNA™	Body suit	Converts heat to IR which expands blood vessels for greater blood flow	Los Angeles, CA
Athos	Athos Wearables	Vest and pant	Muscle activity and heart signals via EMG	San Francisco, CA
Hexo Skin	Astroskin, Smart Kit	Sleeve	Cardiac frequency, respiratory rate and volume, sleep, acceleration	Montreal, Canada
				San Francisco, CA
Huawei	Honor Band A1	Watch	Cardio-respiratory fitness	Shenzen, China
Humon	Hex	Device unit	Non-invasive measurement of O_2_ content in muscles	Boston, MA
Komodo Technologies Inc.	AIO Smart Sleeve	Sleeve	ECG, heart rate, sleep analysis	Winnipeg, Canada
Kymira	Kymira Sports	T-Shirt	Smart garments for cardiac monitoring to prevent heart attacks in athletes	Reading, United Kingdom
LifeBeam	LifeBeam baseball cap	SmartHat	Embedded sensors measure heart rate and calories	New York, NY
MC10	BioStamp RC™, BioStamp nPoint®, Kintinuum	Epidermal sensor	BioStamp RC™: activity, cardiac activity, EMG, and posture	Boston, MA
			BioStamp nPoint®: activity, posture EMG, and sleep metric, vital signs	
			Kintinuum: quantify treatment efficacy	
Myovolt	Myovolt	Sleeve	EMG to increase circulation, boost muscle power	Hong Kong, China
Rotex Technologies	roMage, roSport, roCare, roFashion	Electronic tattoo	roMage: brain and muscle control, gesture recognition	Austin, TX
			roSport: blood O_2_ saturation, heart rate, skin hydration	
			roCare: blood pressure, ECG, respiration, skin hydration, temperature	
			roFashion: glows on skin (fashion purposes)	
Spire	Spire	Device unit	Measures respiration to quantify and detect stress, calorie tracking, pedometer	San Francisco, CA
Withings	Steel HR, Activité, Go, Pulse O_2_	Bands	Heart rate, distances, email, text messages, phone	Issy-les-Moulineaux, France
Xiaomi	Mi Band	Band	Time, pedometer, heart rate	Beijing, China
Xmetrics	Xmetrics Pro, Xmetrics Fit	Device unit	Calories, strokes taken, laps swam, pace	Milan, Italy

Data for this table was acquired from company websites and social media sites affiliated with each company

**Table 5:** Examples of wearable technology companies for monitoring sleep

Company	Sampling of products	Product type	Product functionality	Headquarters
Emfit	Emfit QS	Device unit	Tracks sleep by monitoring movement and heart rate	Vaajakoski, Finland
Kokoon	Kokoon	EEG Headphones	Movement and EEG sensors determine relaxation and sleep quality	Limerick, Ireland
Moov	Moov	Wrist-based device	Heart rate, sleep quality, and activity tracker	San Francisco, CA
WHOOP	WHOOP Band	Wrist-based device	Heart rate, body temperature, movement, and sleep	Boston, MA

Data for this table was acquired from company websites and social media sites affiliated with each company

## Physical performance and safety of the athlete

### Position and motion

**Fig. 2:**
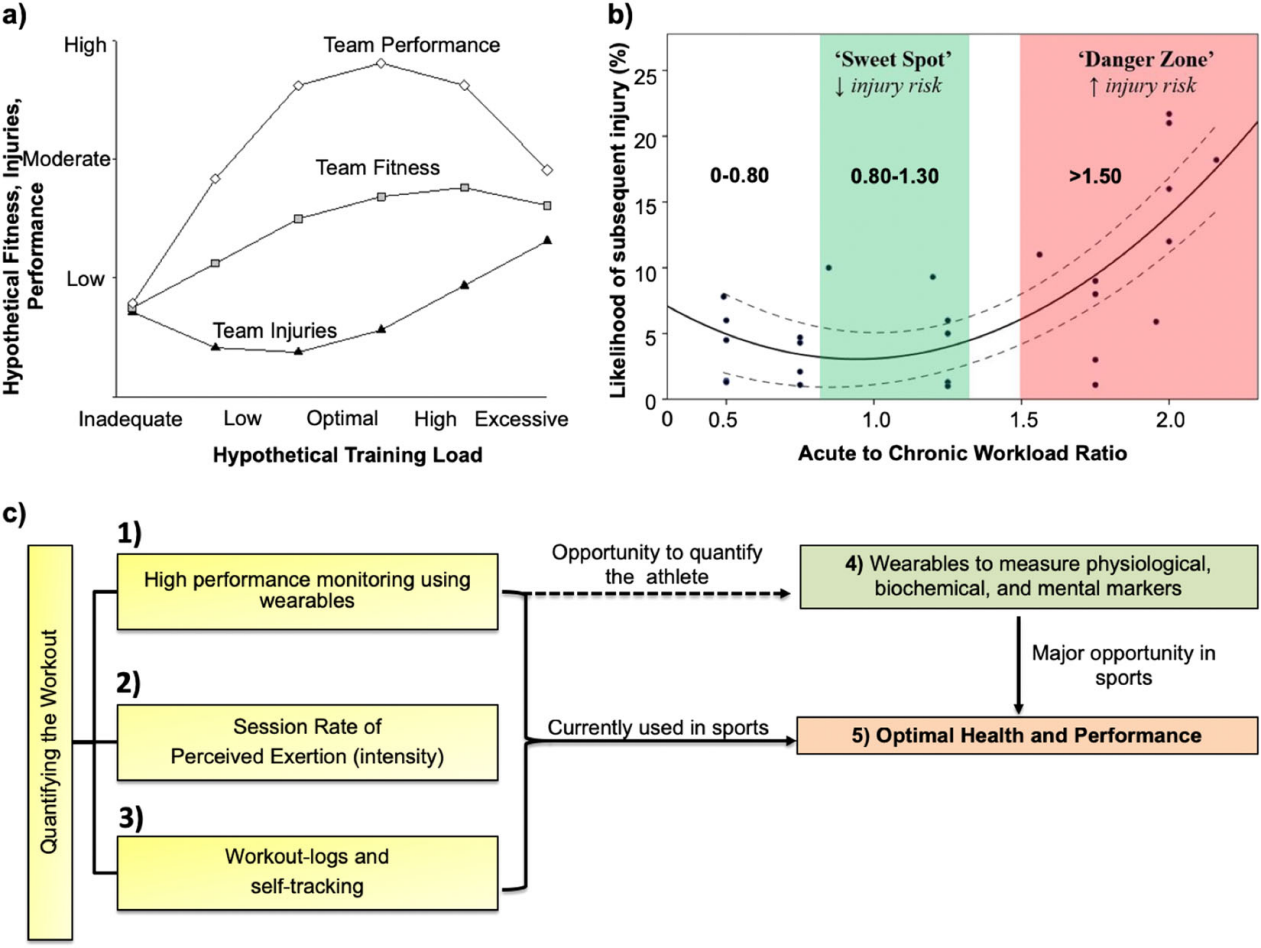
Value proposition of wearable sensor technology to monitor athlete training load to minimize soft-tissue injuries. **a** Hypothetical relationship between training loads, fitness, injuries, and performance. Inadequate and excessive training loads could result in increased injuries, reduced fitness, and poor team performance. **b** Interpreting and applying ACWR data to predict the likelihood of subsequent injury. The green-shaded area (‘sweet spot’) represents the ACWR where the risk of injury is low. The red-shaded area (‘danger zone’) represents the ACWR where the risk of injury is high. To minimize the risk of injury, athletes should aim to maintain their ACWR within a range of ~0.8–1.3. **c** Athlete workout can be monitored via workout logs and self-tracking methods, assessing the sRPE levels, or using wearable technology to quantify movement parameters. The application of wearable sensors to monitor athletic performance and training has provided an added advantage compared to current and past methods by enabling sports scientists and clinicians to quantify the workout, to calculate the ACWR, and to predict the onset of injury. Figure was adapted and modified from Gabbett et al. ^46^ **a**, **b**

The ability to monitor position and movement profiles of an athlete is critical in developing improved training regimens to maximize individual performance (Fig. 2). The accuracy of devices, such as pedometers has been in question and was recently studied. Researchers compared the accuracy of the “step-count” feature between dedicated smartphone-based pedometer applications (Galaxy S4 Moves App, iPhone 5s Moves App, iPhone 5s Health Mate App, iPhone 5s Fitbit App) and wearable devices (Nike Fuelband, Jawbone UP24, Fitbit Flex, Fitbit One, Fitbit Zip, and Digi-Walker SW-200) with direct observation of step counts.^28^ Results showed a relative difference between actual and reported mean step count of −0.3% to 1.0% for pedometers and accelerometers, −22.7% to −1.5% for wearable devices, and −6.7% to 6.2% for smartphone applications. Such differences were attributed to the robustness of the IC technology and software algorithms used to determine a step. Step counts are often used to derive other measures of physical activity, such as distance traveled or calories expended.^28^ Hence, improving measurement accuracy is crucial to measure and appropriately tailor workout regiments for elite-level athletes.

Movement-based sensors currently in use for sports-medicine include accelerometers and global positioning satellite (GPS) devices, often used in combination (Table 1). Accelerometers generate highly accurate analyses of movement with high sampling rates and have been included in wrist-based devices, such as the Nike Fuelband, Jawbone UP, and Microsoft Band. This technology has been widely adopted in the sporting community ranging from Australian Football,^29^ Rugby,^30,31^ NFL,^32^ National Hockey League (NHL),^33^ and swimming.^34–36^ Energy expenditure can be determined from tri-axial accelerometers via the integration of acceleration over time.^37,38^ The determination of energy expenditure, position, movement, and balance control during practices or games has shown to be instrumental in tailoring the training regimen of athletes to minimize the incidence of soft tissue injuries.

Banister et al. postulated that athletic performance can be estimated as a function of fatigue and fitness^39^ (Fig. 2). Building upon this model, Morton et al. suggested that an opportune training stimulus is one that maximizes performance by utilizing an appropriate training load, while simultaneously minimizing injury and fatigue.^40^ A working definition of fatigue is “any exercise-induced or non-exercise-induced loss in total performance due to various physiological factors, athlete reported psychological factors, or a combination of the two”.^41^ It is well known that fatigue decreases athletic performance and that training induces numerous neurophysiological and psychological changes in an athlete’s body. There are two forms of fatigue: central fatigue and peripheral fatigue. Central fatigue is the fatigue resulting from the central nervous system (CNS) and the transmission of signals from the brain to the muscle.^42^ Central fatigue is related to the interaction between the brain and the spinal cord.^43^ Researchers have hypothesized that the differentiation between a good athlete and an elite-level athlete is their individual ability to ignore such neurotransmissions during high-acuity situations (e.g. high profile matches or workouts).^42^ Peripheral fatigue is the failure to maintain an expected power output caused by the depletion of glycogen, phosphate compounds, or acetylcholine within the muscular unit or by the accumulation of lactate or other metabolites that are released during activity.^44,45^ Peripheral fatigue occurs within the muscle and can be thought of as ‘muscle fatigue’.^43^ As such, wearable sensors can be used to measure parameters indicative of the peripheral fatigue of the athlete, as is discussed in detail throughout this review. For simplicity purposes, we refer to peripheral fatigue as simply fatigue.

Monitoring internal (e.g. physiological or perceptual ‘response’) and external training loads (e.g. physical ‘work’) can enable sports trainers and clinicians to assess the fatigue and fitness levels of athletes in real time. Internal workload includes the session rate of perceived exertion (sRPE) and heart rate.^46^ At the completion of each training session, athletes provide a 1–10 ‘rating’ based on the intensity of the session.^46^ The intensity of the session is multiplied by the session duration to provide the internal training load.^46^ The product can be thought of as the athletes’ “exertional minutes”.^46^ Advancements in MEMS fabrication techniques and device packaging have allowed for the detection of multi-axial movement to calculate an external training load (e.g. PlayerLoad™^3^). External workload can be thought of as how much load is placed on the body and can be quantified using torso-mounted wearable devices which contain a GPS and a tri-axial accelerometer.^46^ PlayerLoad™ can be calculated via the instantaneous rate of change of acceleration. Accumulated PlayerLoad™ can be calculated as the summation of PlayerLoad™ over the desired time interval (usually over a span of 1–7 days).

**Fig. 3:**
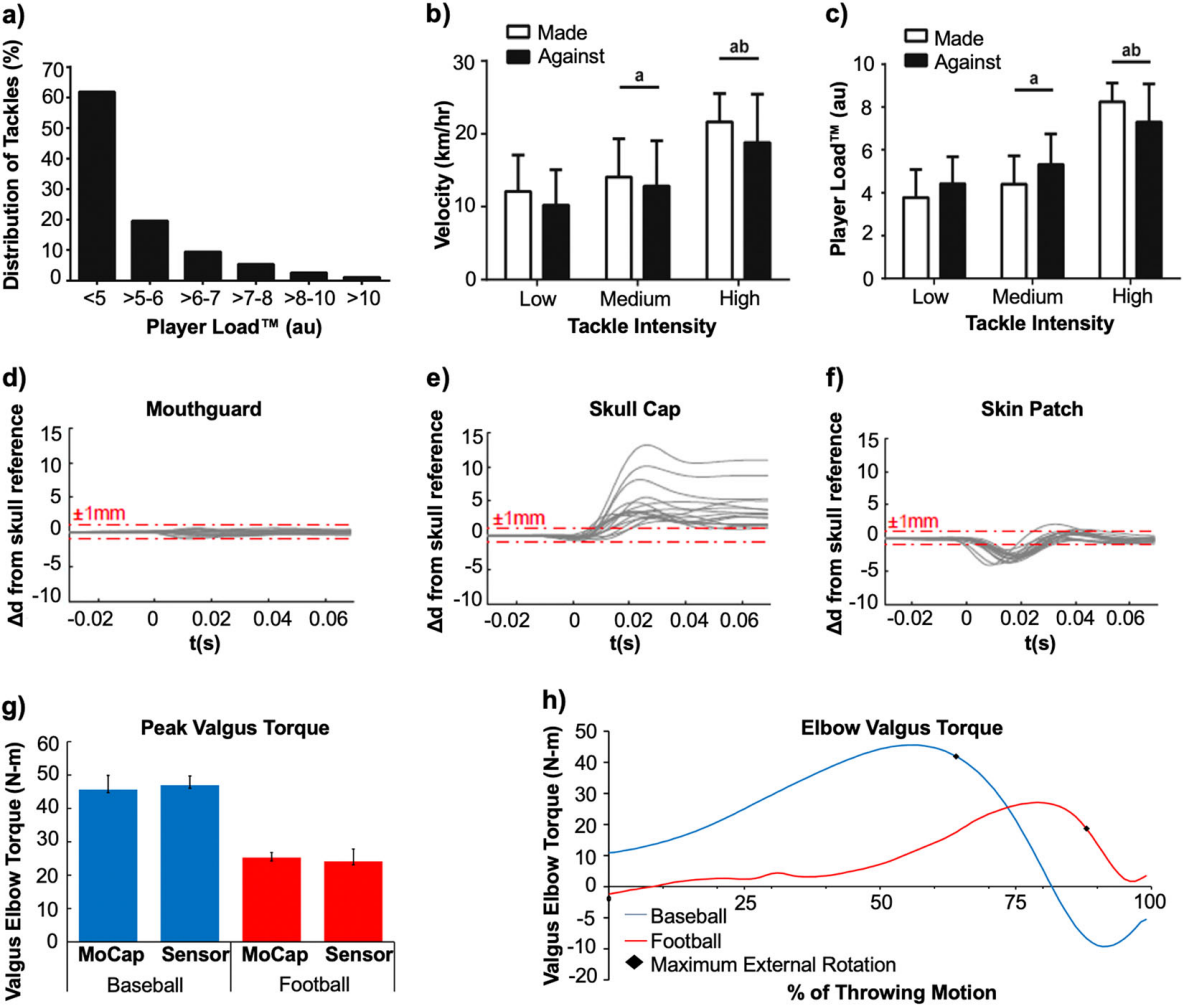
Wearable sensors monitor the biomechanical performance of the athlete. **a** Distribution of tackles (*n* = 352) made and against peak instantaneous Player Load™. **b** Peak velocity for tackles made and against associated with tackle intensity categorized as low (*n* = 115), medium (*n* = 216), and high (*n* = 21). **c** Peak Player Load™ for tackles made and against associated with tackle intensity categorized as low (*n* = 115), medium (*n* = 216), and high (*n* = 21). **d** Relative displacements of the mouthguard sensor from the skull studied using high speed video. Among 16 trials, the mouthguard always had the smallest (sub-millimeter) displacement from the skull, within video error, compared to the skull cap and skin patch. **e** Relative displacements of the Reebok skull cap from the skull studied using high speed video. **f** Relative displacements of the xPatch Gen2 skin patch sensor from the skull studied using high speed video. **g** motusBASEBALL sensor exhibited higher peak elbow valgus torque for baseball pitching compared to football throwing. Data demonstrates the utility of the sensor to measure biomechanical forces during non-stationary periods on an athlete. **h** motusBASEBALL sensor used to quantify the average valgus torque on the elbow for baseball pitching and football throwing between foot contact and maximum internal rotation. “^a^Significantly different (*p* < 0.01) from Low; ^b^significantly different (*p* < 0.01) from Medium. No significant differences between tackles made and against.” Figures were reproduced with permission from Gastin et al.^29^ **a**–**c**, Wu et al.^79^ **d**–**f**, and Laughlin et al.^89^ **g**–**h**

Metrics such as total distance run, weight lifted, number and intensity of sprints or collisions can be determined using GPS-based sensors. Position sensors triangulate signal transmission from multiple GPS satellites orbiting the earth and can accurately determine the velocity and position (within 1 m) of an athlete on a field. These devices are playing an instrumental role in sports performance analysis by allowing coaches, physicians, and trainers to better understand real-time physical demands of an athlete.^30,37,47^ GPS silicon chips combined with tri-axial accelerometers have been used to record physical activities during different times of the day and for specific position groups on a team.^48^ The majority of work to assess human motion and its correlation to sports performance has involved the use of commercial GPS-based devices, such as the Catapult devices (OptimEye S5) and Zebra Technologies GPS device. The Catapult product, for example has a fully packaged processing IC, accelerometer, gyroscope, and magnetometer to measure body position, impact forces, velocity, acceleration, and direction in a continuous manner.^49^ In a study utilizing the Catapult OptimEye S5 and video tracking technology, 20 professional Australian Football League (AFL) players were studied during four in-season matches to describe and quantify the frequency, velocity, and acceleration at impact during tackling^29^ (Fig. 3a–c). Distributions in tackles were quantified and classified as a function of percent distribution of tackles versus player load (Fig. 3a), player velocity versus tackle intensity (Fig. 3b), and player load versus tackle intensity (Fig. 3c). Differences in accelerometer data between tackles were observed to be progressively greater in intensity thereby providing support for the use of accelerometers to assess impact forces in contact-based sports.^29^ In another study, GPS sensors and related analytics were used by National Collegiate Athletic Association (NCAA) Division I Football athletes to record workload, velocity, distance, and acceleration during both practices and games.^48,50^ The studies found significant variation in movement profiles among collegiate football players and the authors identified the need for position-specific and game-specific physical conditioning strategies to maximize player performance, limit the effects of fatigue, and minimize the onset of injuries.

The combination of the internal and external workloads of the athlete determine the training outcome.^46^ An athlete’s internal or external workload can be computed over a 1-week period (acute workload) and over a 3–4-week period (chronic workload). Work by Gabbett suggested that the ratio of the acute-to-chronic workload, herein referred to as “ACWR”, can be used to determine if an athlete is overtraining, undertraining, or training at the opportune intensity^46^ (Fig. 2b). Furthermore, Gabbett showed that calculation of this ratio enables sports scientists to predict the chance an athlete suffers an injury as a result of improper load management.^46^ Deriving this ratio provides an index of athlete preparedness and considers the training load that the athlete has performed relative to the training load that the athlete has been prepared for.^51^ The use of the ACWR emphasizes both the positive and negative consequences of training. The first study to investigate the relationship between ACWR and the risk of injury was performed on elite cricket fast bowlers.^52^ Training loads were estimated from both sRPE and balls bowled. When the acute workload was similar to or lower than that of the chronic workload (e.g. ACWR ≤ 0.99), the likelihood of injury for fast bowlers in the next 7 days was 4%.^52^ However, when the ACWR was ≥1.5 (e.g. workload in the current week was 1.5 times greater than what the bowler was prepared for), the risk of injury was 2–4 times greater in the subsequent 7 days.^52^ While such observations are indicative of the sport being studied, until more robust data sets are available, caution must be heeded when applying these recommendations to individual sport athletes. Despite this, a general trend can be concluded. If the acute training load is low (e.g. the athlete is experiencing minimal fatigue) and the rolling average (RA) chronic training load is high (e.g. the athlete has developed ‘fitness’), then the athlete will be in a well-prepared state and thus, the ACWR will be ≤1.^46^ If the acute load is high (e.g. training loads have been rapidly increased resulting in ‘fatigue’) and the RA chronic training load is low (e.g. the athlete has performed inadequate training to develop ‘fitness’), then the athlete will be in a fatigued state and thus, the ACWR will be ≥1.^46^ In terms of injury risk, ACWRs within the range of 0.8–1.3 could be considered the training ‘sweet spot’, while an ACWR ≥ 1.5 could represent the ‘danger zone’.^46^

The RA model^53^ (Eqs. (1–4)) and exponentially weighted moving average (EWMA) model^54^ (Eqs. (5–10)) are two methods used to calculate the training load of the athlete with or without the use of wearable sensors like the Catapult OptimEye S5 (Eqs. (11–14)).^3^ The RA model uses an absolute (i.e. total) workload performed in one week (acute workload) relative to the 4-week chronic workload (e.g. 4-week average acute workload).^53^ Equation (1) represents the exertional minutes per workout which is the product of the session rating of perceived exertion and the duration of the workout in minutes. The sRPE is a scale from 1 to 10 with progressing intensity of the workout deemed by the athlete and training staff. Equation (2) shows the acute player load (PL) which is the summation of the exertional minutes per workout for a given week (e.g. from day 1 to day 7). For the sake of simplicity, we assume the athlete is completing one workout per day. Equation (3) shows the chronic PL which is computed by taking the average of the acute PL over the duration of weeks (denoted as *n*). Equation (4) shows the ACWR which is the ratio between the acute PL for the given week (Eq. (2)) and the chronic PL (calculated from Eq. (3)). The RA model suggests that each workload in an acute and chronic period is equal. In other words, the model considers there to be a linear relationship between load and injury. The assumption in this model is that all workload in a given time period is equivalent. Key drawbacks of this model are that the model does not account for any decays in fitness and it does not accurately represent variations in the manner in which loads are accumulated.

1 $${\mathrm {Exertional}}\;{\mathrm {minutes}}\;{\mathrm {per}}\;{\mathrm {workout}} = \left( {{\mathrm {SRPE}}} \right) \times \left( {{\mathrm {duration}}\;{\mathrm {of}}\;{\mathrm {workout}}\;{\mathrm {in}}\;{\mathrm {minutes}}} \right)$$

2 $${\mathrm {Acute}}\;{\mathrm {PL}} = \mathop {\sum}\nolimits_{D = 1}^{D = 7} {{\mathrm {exertional}}\;{\mathrm {minutes}}\;{\mathrm {per}}\;{\mathrm {workout}}}$$

3 $${\mathrm {Chronic}}\;{\mathrm {PL}} = \frac{{\mathop {\sum }\nolimits_{W = 1}^{W = n} {\mathrm {Acute}}\;{\mathrm {PL}}}}{n}$$

4 $${\mathrm {ACWR}} = \frac{{{\mathrm {Acute}}\;{\mathrm {PL}}\;{\mathrm {for}}\;{\mathrm {given}}\;{\mathrm {week}}}}{{{\mathrm {Chronic}}\;{\mathrm {PL}}}}$$

Sports scientists have started to apply the EWMA model to circumvent the drawbacks posed by the RA model.^54^ The EWMA model places a greater weight on the most recent workload an athlete has performed by assigning a decreasing weighting for each older workload value (time decay constant, *λ*_a_) and the non-linear nature of injury occurrence and workload.^54^ Equation (5) shows the exertional minutes per workout which is the product of the session rating of perceived exertion and the duration of the workout in minutes. The sRPE is a scale from 1 to 10 with progressing intensity of the workout deemed by the athlete and training staff. Equation (6) shows the degree of decay, *λ*_a_, which is a value between 0 and 1, with higher values of *λ*_a_ discounting older observations in the model at a faster rate. In the following equation, *n* represents the time decay constant. Equation (7) shows the formula to calculate the EWMA for a given day which is based on the exertional minutes, calculated from Eq. (5), the degree of decay from Eq. (6), and the EWMA from the preceding day. Equation (8) shows the acute player load (PL) which is the summation of the EWMA for a given week (e.g. from day 1 to day 7). For the sake of simplicity, we assume the athlete is completing 1 workout per day. Equation (9) shows the chronic PL which is computed by taking the average of the acute PL over the duration of weeks (denoted as *n*). Equation (10) shows the ACWR which is the ratio between the acute PL for the given week (Eq. (8)) and the chronic PL (calculated from Eq. (9)). A recent study sought to investigate if any differences existed between the RA and EWMA models pertaining to ACWR calculation and subsequent injury risk in elite Australian footballers.^54^ Fifty-nine athletes from an AFL club participated in this 2-year study. A total of 92 individual sessions were recorded. Each season consisted of a 16-week preseason phase comprised of both running and football-based sessions, followed by a subsequent 23-week in-season competitive phase. The Catapult OptimEye S5 GPS sensor, sampled at 10 Hz, was used to quantify training and match workloads of players. The triaxial accelerometer, gyroscope, and magnetometer were each sampled at 100 Hz. The study demonstrated that a high ACWR was significantly associated with an increase in injury risk for both models. The EWMA model had significantly greater sensitivity to detect increases in injury likelihood at higher ACWR ranges during both the preseason and in-season periods. The study concluded that the EWMA model may be better suited to modeling workloads and injury risk than the RAs than the ACWR model. Regardless of the ACWR model utilized, spikes in acute workload were significantly associated with an increase in injury risk.

5 $${\mathrm {Exertional}}\;{\mathrm {minutes}}\;{\mathrm {per}}\;{\mathrm {workout}} = \left( {{\mathrm {SRPE}}} \right) \times \left( {{\mathrm {duration}}\;{\mathrm {of}}\;{\mathrm {workout}}\;{\mathrm {in}}\;{\mathrm {minutes}}} \right)$$

6 $$\lambda _{\mathrm {a}} = \frac{2}{{N + 1}},{\mathrm{where}}\,0 < \lambda _{\mathrm {a}} < 1$$

7 $${\mathrm {EWMA}}_{{\mathrm {today}}} = \left( {{\mathrm {Exertional}}\;{\mathrm {minutes}}\;{\mathrm {per}}\;{\mathrm {workout}}} \right)\left( {\lambda _{\mathrm {a}}} \right) + \left[ {\left( {1 - \lambda _{\mathrm {a}}} \right)\left( {{\mathrm {EWMA}}_{{\mathrm {yesterday}}}} \right)} \right]$$

8 $${\mathrm {Acute}}\;{\mathrm {PL}} = \mathop {\sum}\nolimits_{D = 1}^{D = 7} {{\mathrm {EWMA}}_{{\mathrm {today}}}}$$

9 $${\mathrm {Chronic}}\;{\mathrm {PL}} = \frac{{\mathop {\sum}\nolimits_{W = 1}^{W = n} {{\mathrm {Acute}}\;{\mathrm {PL}}} }}{n}$$

10 $${\mathrm {ACWR}} = \frac{{{\mathrm {Acute}}\;{\mathrm {PL}}\;{\mathrm {for}}\;{\mathrm {given}}\;{\mathrm {week}}}}{{{\mathrm {Chronic}}\;{\mathrm {PL}}}}$$

Wearable sensors are currently being used to minimize injury in professional football via careful monitoring of training load and other biometrics during the rehabilitation period (Fig. 2c). The variability of GPS data and accelerations of the torso have been in question when it comes to monitoring the loads of the lower limbs. This is because distance traveled and velocity do not represent the mechanical load experienced by the musculoskeletal tissue. This is specifically relevant in sports such as basketball, which are constrained to a small-space, where players experience high loads of physical stress by performing explosive jumping and landing activities, which are not accurately captured by distance, speed, or torso athlete movement analysis systems.^55,56^ To mitigate such issues, the Zebra GPS device and Catapult OptimEye S5, both of which are considered the most accurate wearable devices in sports today, are housed in player tracking devices in an attempt to negate some of the aforementioned issues. Additionally, the Catapult device has shown to mitigate such issues by incorporating tri-axial movements into their analytical models to accurately calculate PlayerLoad™ from their sensor.^3^ The Zebra GPS device is currently approved by the NFL for use to track player movement and has been utilized by select teams to monitor training loads.^57^ Equation (11) provides the analytical platform of the Catapult OptimEye S5 which utilizes a tri-axial accelerometer to calculate PlayerLoad™ (PL) based on acceleration in the *x*, *y*, and *z* directions. Equation (12) shows the summation of PL from the initial to end time of interest (in most cases this is from the start to the end of a training session) denoted as AccPL™. Equation (13) shows how the RA model can be used to calculate Acute PL, analogous to Eq. (2). However, in this case, PL is calculated from Eq. (11) using a wearable sensor. Eq. (14) shows how the EWMA model can be used to calculate PL for the given day using PL calculated from a wearable sensor. The ACWR can be calculated utilizing either model, adapting the set of equations presented (rolling average, Eqs. (1–4); EWMA, Eqs. (5–10).

11 $${\mathrm {PL}} = \sqrt {\left( {{\mathrm {fwd}}_{t = i + 1} - {\mathrm {fwd}}_{t = i}} \right)^2 + \left( {{\mathrm {side}}_{t = i + 1} - {\mathrm {side}}_{t = i}} \right)^2 + \left( {{\mathrm {up}}_{t = i + 1} - {\mathrm {up}}_{t = i}} \right)^2}$$

12 $${\mathrm {AccPL}} = \mathop {\sum}\nolimits_{t = 0}^{t = n} {\sqrt {\left( {{\mathrm {fwd}}_{t = i + 1} - {\mathrm {fwd}}_{t = i}} \right)^2 + \left( {{\mathrm {side}}_{t = i + 1} - {\mathrm {side}}_{t = i}} \right)^2 + \left( {{\mathrm {up}}_{t = i + 1} - {\mathrm {up}}_{t = i}} \right)^2} }$$

13 $${\mathrm {Acute}}\;{\mathrm {PL}} = \mathop {\sum}\nolimits_{D = 1}^{D = 7} {{\mathrm {AccPL}}}$$

14 $${\mathrm {EWMA}}_{{\mathrm {today}}} = \left( {{\mathrm {PL}}} \right)\left( {\lambda _{\mathrm {a}}} \right) + \left[ {\left( {1 - \lambda _{\mathrm {a}}} \right)\left( {{\mathrm {EWMA}}_{{\mathrm {yesterday}}}} \right)} \right]$$

In a specific example reported by an American sporting network, the device was used to accurately track the recovery of an athlete after the individual suffered a season ending injury the previous year.^57^ The sensor was placed underneath the shoulder pads (analogous to that of the Catapult device) or sewn into the jersey to generate biometric measurements, such as movement profiles and workload to gauge the athlete’s performance and workload during recovery relative to his peak performance and workload prior to the injury. Additionally, utilizing the Catapult OptimEye S5 wearable sensor, authors of this review have recently studied the effects of player workload on soft tissue injuries in a single NFL team over two seasons.^32^ Rapid changes in workload over a one-week period when compared to the average workload over a month were associated with a significant increase in risk of hamstring and other soft tissue injuries. The studies demonstrated that monitoring athletic training programs during the pre-season compared to the post-season utilizing wearable technology have assisted team athletic trainers and medical staff in developing programs to optimize player performance and minimize soft-tissue injuries.^32^

### Impact detection

The spongy nature of a woodpecker’s skull acts like a shock absorber by pinching the jugular vein to increase blood pressure in the brain to protect it from the 12,000 daily hammerings it performs on trees.^58^ Unfortunately, humans do not have any sort of ‘protection mechanism’ to mitigate or dissipate impact forces on the brain.^58^ The onset of concussions, brain injuries, and mental health illnesses caused by repeated trauma to the head have paved the way for newer technologies to detect and eliminate chronic traumatic encephalopathy (CTE).^59^ CTE is a neurodegenerative disease found in individuals who have experienced repeated traumatic brain injury (TBI) or concussions. In these conditions, stretching, compression, and shearing of axons during sudden brain movements over extended periods are hypothesized to cause axonal injury.^60^ The high incidence of such injuries in athletes is of major concern in modern collision sports.^61^ The American Academy of Neurology (AAN) defines a concussion as a “pathophysiologic disturbance in neurologic function characterized by clinical symptoms induced by biomechanical forces”.^62^ Guskiewicz et al. concluded that former NFL and collegiate football players who reported multiple concussions were at higher risk for depression and memory loss.^63,64^ Research on concussions and CTE is still rudimentary and primarily supported by clinical models.^59^ There remains a strong clinical need to develop devices, which could quantitatively and qualitatively measure impact forces on the brain to decrease the onset of concussions and reduce the incidence of CTE. Currently, work is being done to design custom personal protection equipment (PPE), such as helmets and mouthguards to improve player safety.^65^ Research by Stenger et al. and McCrory et al. showed the potential applicability of mouth guards towards preventing head and spinal injuries.^66,67^ Companies such as i1 Biometrics, Mamori, and Force Impact Technologies have developed mouthguards that can monitor concussions (Table 2). The mouthguard by Force Impact Technologies contains embedded sensors, which relate collision intensity using color-coded LED’s (green, blue, or red) located at the front of the mouthguard. The colors are representative of the impact force delivered; green represents a mild impact, blue represents a medium impact force, and red represents a major impact force. The displayed color is then relayed via Bluetooth to the appropriate medical personnel in order to initiate the necessary protocols and interventions. The company believes the sensor placement will provide a high correlation back to the center of the brain. Despite the potential benefit of this technology, mouthguards are not universally used by all athletes. There remains a need to design and fabricate wearable sensors that can monitor and quantify impacts during collisions.^68^

Several wearable device companies such as Noggin, Q30 Innovations, and X2 Biosystems have gained prominence in their ability to track, monitor, and prevent concussions. Noggin is focused on creating a protective skull cap whereby a gel cap generates friction with the inside of the helmet to hold it in place. This reduces slippage while dispersing and reducing impact forces on the head.^69^ The device also has a dry moisture wicking fabric that helps to protect athletes from heat-induced injury.^69^ Noggin has shown that its device can decrease impact forces up to 85% via a direct blow to the head when used with an approved helmet.^69^ Inspired by the woodpecker, Q30 innovations designed a device that prevents the brain from moving within the skull by clamping down on the jugular veins, causing the brain to swell and fit more snugly within the skull.^70^ Myers et al. tested the Q-collar device on youth hockey and high-school football players and successfully demonstrated that using the wearable device during live-game scenarios may have provided a protective barrier against the microstructural changes of the brain caused after repetitive head impacts.^71,72^ The studies used helmet accelerometers to track the number of hits a player received that had accelerations >20 g. Magnetic resonance imaging (MRI) was used to qualitatively observe and measure the diffusivity of water in different parts of the brain prior to and after the study. Although this device has not yet received FDA approval, it shows tremendous promise in reducing the incidence of concussions and TBI in collision athletes. The X-Patch Pro wearable sensor and X2 Mouthguard devices by X2 Biosystems are currently the most utilized head impact measuring devices in the sports community.^73^ The X-Patch Pro is an epidermal sensor containing an adhesive that can be worn behind the ear to record head impacts. The device transmits to a sensor data management (SDM) application on an electronic device.^74^ The sensor demonstrated a reduction in the incidence of head impacts leading to a decrease in concussions by 30–70% and is currently being utilized to study cumulative brain damage due to repeated head impacts.^74^ The sensors contain a tri-axial high-impact linear accelerometer and a triaxial gyroscope to capture six degrees of freedom for linear and rotational accelerations.^73,74^ X2 Biosystems utilizes proprietary analytical software called xSposure to relate acceleration measurements with impact duration, ranked from 1 (mild impact) to 10 (major impact).^75,76^ Additionally, the device calculates a Gadd Severity Index (GSI), head impact telemetry severity profile (HITsp), and generalized acceleration model for brain injury threshold (GAMBIT). Collegiate football teams at the University of Virginia^75^ and the University of Mississippi^76^ have utilized wearable devices by X2 Biosystems. Recently, professional football teams have adopted this device to monitor and track their own players.^77^ Research by the University of Virginia on their NCAA Division I-A football team compared the number and severity of sub-concussive head impacts sustained during helmet-only practices, shell practices, full-pad practices, and live-game scenarios to determine whether sub-concussive head impact on college athletes varies with practice type.^75^ The 20 participating football players wore the xPatch impact-sensing skin patches on the skin covering their mastoid process over the course of a season. Results showed that regulation of practice equipment could offer a viable and promising solution to drastically reduce sub-concussive head impact in collegiate football players. In another study, the University of Mississippi utilized the xPatch skin sensor to monitor head impacts on 15 NCAA Division I-A football players.^76^ After each practice, players reviewed their head impact profiles to determine the correlation between their head impacts relative to tackling technique and form. Results showed that the xSposure score of these players decreased by 15% over the course of the preseason.^76^ Wu et al. utilized high speed video to test a teeth-mounted mouthguard (developed by the research group in a previous study^78^), X-Patch Gen 2 soft tissue-mounted patch (adhered to the skin on the mastoid process), and the Reebok elastic skull cap during sagittal soccer head impacts (Fig. 3d–f).^79^ The study focused on a 26-year-old male human subject who underwent soccer head impacts with clenched teeth. The ball traveled at an initial velocity of 7 m/s and was inflated to 8–9 psi.^79^ This velocity represented the average header speed in youth soccer. The researchers developed a method to quantify skull coupling of wearable head impact sensors in vivo. Furthermore, they found that in-plane skin patch acceleration peaked in the anterior–posterior direction and could be modeled by an underdamped viscoelastic system. The mouthguard showed tighter skull coupling than the other sensor mounting approaches (Fig. 3d–f). Additionally, the skin patch and skull cap had higher displacements from the skull compared to the mouthguard (Fig. 3d–f). Results from these studies demonstrated that wearable devices can track and minimize concussions; however, further clinical trials and a more in-depth understanding of the analytical platforms and modeling of sensor performance are needed to have a true clinical impact in sports. The work by Reebok is particularly interesting as it entails a partnership with MC10, a start-up originally out of the Rogers research group. The Reebok Checklight includes one or more accelerometers wired up with MC10’s “stretchable” electronics which consist of ultrathin gold electrodes that match the contour of the body.^80^ The partnership highlights the successful translation of scientific research into a commercial product to monitor impact forces on the head in a real-time manner.^80^ In another study, researchers developed a dry, textile-based nanosensor that detected early signs of TBI by continuously monitoring various neural behaviors indicative of the injury, such as drowsiness, dizziness, fatigue, sensitivity to light, and anxiety.^81^ The device comprised of a network of flexible sensors woven or printed into a skullcap worn underneath a football helmet. The device used Zigbee/Bluetooth wireless telemetry to relay the data from the sensors to a receiver and to a remote monitor. The system included a pressure-sensitive textile sensor embedded underneath the helmet's outer shell, which measured the intensity, direction, and location of the impact force. The other sensors worked as an integrated network within the skullcap and included a printable and flexible gyroscope that measured rotational motion of the head and body balance and a printable and a flexible 3-D accelerometer that measured lateral head motion and body balance. Additionally, the device was outfitted with physiological sensors to detect pulse rate and blood oxygen levels. At time of publishing, the device had yet to be tested in real-time football games. While follow-up clinical data is not available, assessing the use-case of such devices in randomized, controlled studies is necessary to further translate such technology to improve athletic safety and performance.

### Biomechanics detection

Motion analysis to study biomechanics is currently performed by measuring body kinematics via motion capture systems such as optical, inertial or magnetic units (IMU), electrogoniometers, and mechanical tracking.^82^ However, their disadvantages prevent them from being utilized for an extended duration to monitor human movement. Optical systems are expensive, require complex setups, and data processing systems, and are rarely used to assess a single joint or body part. IMU-based systems have limited fields of operation, high error rates, and high sensitivities. Mechanical tracking systems have poor portability and cannot be used during real competition situations. Therefore, there is a strong need to develop alternative technologies to monitor and quantify human-body kinematics in a non-invasive and accurate manner.

Epidermal wearable sensors can play a key role in quantifying human movement and monitoring changes in joint mechanics in order to prevent injuries. Key properties of these sensors for biomechanical detection include their high stretchability, flexibility, robustness, and durability.^9,83,84^ These sensors can be applied over the joint as a sleeve to monitor the stress and strain on the elbow, anterior cruciate ligament (ACL), medial collateral ligament (MCL), or posterior cruciate ligament (PCL). In 2015, 25% of active major league baseball (MLB) pitchers and 15% of minor league pitchers underwent ulnar collateral ligament (UCL) reconstruction. Often referred to as Tommy John surgery,^85^ the UCL in the medial elbow is replaced with either an autograft or donor allograft tendon. This reconstructive surgery typically sidelines a pitcher for the entire season due to the time-intensive rehabilitation that follows.^86^ Data from Motus Global in 2015 showed that pitchers represented ~59% of injured players in MLB and $420 million in sidelined salaries.^85^ The “motusBaseball Sensor,” developed by Motus Global, is the first wearable device approved by the MLB for in-game use.^87^ The wearable sleeve quantifies the strain exerted by a pitcher to gain a better understanding of the factors that cause ligament damage.^88^ The device contains five sensors and an analytical program to view the biomechanical data. A single sensor near the elbow measures the stress exerted on the UCL. The sensor also has clinical utility for football quarterbacks (QBs). Football quarterbacks exhibit overarm throwing injuries due to overuse and require rehabilitation therapy from injuries on the throwing arm caused by contact. A recent paper by Motus Global tested the motusBaseball Sensor on a high school male baseball pitcher.^89^ The athlete was instrumented with 46 reflective markers on anatomical locations and kinematic data were collected at 480 Hz using a 12-camera 3D motion capture system (Motion Analysis Corp, mocap). The motusBASEBALL sensor was placed on the inside of the forearm ~3 cm distal to the medial elbow epicondyle.^89^ The athlete pitched a baseball off a mound into a net at a distance of ~5 m away from the pitching rubber. Following this, the athlete made seven throws with a football in a “shotgun” stance (e.g. no drop-back prior to throwing). Full body kinematics were used to calculate elbow valgus torque by both mocap and the motusBASEBALL sensor. The sensor read slightly higher peak elbow valgus torque for baseball pitching (3%) and slightly lower in football throwing (5%) (Fig. 3g).^89^ The results demonstrated that the sensor was successful in calculating maximum elbow valgus torque in both baseball and football throwing scenarios (Fig. 3g).^89^ While statistical analysis was not performed, the authors of the study concluded that the differences between the mocap calculations of torque and sensor calculations of torque were minor. The study showed that the motusBASEBALL sensor provided an accurate measure of elbow valgus torque for both baseball pitching and football throwing (Fig. 3h).^89^ Use of this data from the sensor could enable measures of acute and chronic workloads that are joint specific to the throwing arm to improve performance and minimize injury. We hypothesize that such information could enable coaches to refine throwing techniques to serve as “coachable moments” for athletes specializing in throwing-based sports to minimize serious long-term injury. Detecting biomechanical forces and arm angles have been utilized as teaching tools in sports as well. Vibrado Technologies has developed a wearable sleeve that measures arm angles and movement to model shooting motion in basketball^90^ (Table 3). This device has potential applications in basketball training and other sports where “muscle memory” is crucial for repeated success.

The ACL is the primary stabilizing knee ligament that prevents anterior translation of the tibia.^91^ ACL tears are one of the most common knee injuries observed in sports medicine. Forces in the ACL can be studied and quantified in six degree of freedoms (DOF) due to externally applied loads.^91^ An accurate device to measure the biomechanics to determine the correlation between the ACL and the kinematics of the knee is necessary for the longevity of athletes and for sports trainers and physicians to better tailor rehabilitative therapy for the athlete.^91^ Currently there is no quantifiable method or commercial device to determine ACL strain. Thus, the development of a robust sensor capable of such measurements is highly desirable for cutting and pivoting sports, such as soccer, football, basketball, and rugby.^3^ In a recent study, a wearable inertial-based device to evaluate ACL injury risk during jumping tasks was designed.^92^ The accuracy of the sensor was measured by comparing temporal events (initial contact, toe-off), jump height, and sagittal plane angles (knee, trunk) to simultaneous measurements obtained with a marker-based optoelectronic reference system on 38 healthy subjects. Overall, the wearable system demonstrated good concurrent validity with marker-based measurements and good performance in terms of the known risk factors for ACL injury. However, the obtrusive nature of the device severely hindered its utility for use during team-based activities thus necessitating significant modifications (e.g. miniaturized and unobtrusive form factor) for the athlete.

## Physiological status of the athlete to optimize on-field performance

### Heart rate and electrocardiogram detection

Heart rate (HR) is a key indicator of physiological adaptation, exercise intensity, and workout effort.^93^ Standard HR monitors are comprised of a transducer worn around the chest that can store data locally for 1–2 weeks or alternatively transmit the data to a wireless wrist display.^94^ Newer detection methods, such as photoplethysmography (PPG), utilize optical sensors to detect HR directly from the wrist or fingertip by detecting blood volume changes as a function of transmitted and reflected light. Prior studies showed that analyzing HR data allowed researchers to more accurately quantify physical activity in individuals. On that note, researchers examined the relationship between HR and maximal oxygen consumption (VO_2_) during field and laboratory-based moderate intensity workouts.^93^ Energy expenditure (EE) was estimated from HR data by adjusting age and fitness by expressing the EE data as a percentage of HR reserve (HRR) and a percentage of VO_2_ reserve (VO_2_R).^93^ Results demonstrated that HR was a relatively accurate predictor of EE (*r* = 0.87).

Current HR devices on the market include the Xiaomi mi Band, Apple Watch, Garmin and Fitbit devices, Komodo AIO Smart Sleeve, Jabra Sports Pulse headphone, WHOOP Band, and the Zephyr Bioharness™ (Table 4). The Komodo AIO Smart Sleeve contains a processor and internal memory incorporated within the fabric to collect information about an individual’s HR, sleep quality, and workout intensity. The sleeve contains a conductive liquid that connects one electrode on the user’s wrist to another electrode under the arm.^95^ Clinical validation of this device is needed to assess its efficacy for athletes. The Jabra Sports Pulse headphones have ushered in a new wave of wearable devices referred to as “hearables”. Jabra’s HR sensor lies against the bottom of the inside rim of the left ear canal. Studies from Jabra have shown that HR readings are 99.2% comparable to ECGs.^96^ Additionally, wearable sensors have been designed for the concurrent detection of various physiological parameters. The Zephyr Bioharness™ can simultaneously measure five physiological and activity-related parameters, such as HR, breathing frequency, skin temperature, tri-axial movement, and posture in real-time.^97,98^ The sensor is affixed to clothing via a strap and worn around the abdomen. The Bioharness™ is being utilized for physical activity and exercise monitoring, emergency situations, and for monitoring the well-being of military personnel.^58,99^

**Fig. 4:**
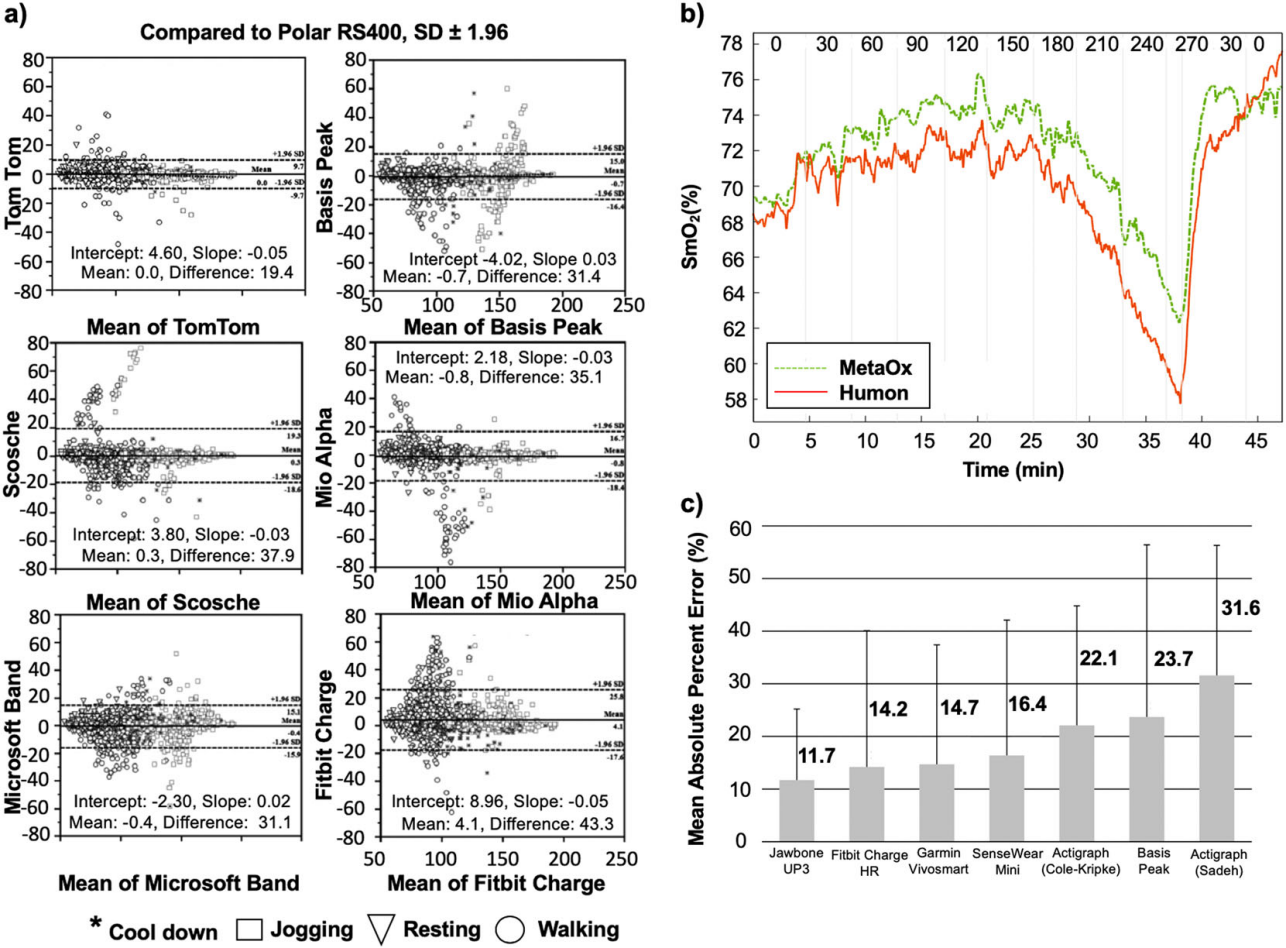
Wearable sensors monitor the physiological status (heart rate, muscle oxygen saturation, and sleep) of the athlete. **a** Bland Altman plots for all wearable wrist-sensors compared to the Polar RS400. *x*-axis: Mean of PolarRS400 and tested device; *y*-axis: PolarRS40 and tested device. **b** SmO_2_ results for a representative subject during an incremental cycling test. The Humon Beta SmO_2_ (red line) and MetaOx SmO_2_ (green line) absolute values are 3–5% different; however, the overall trend holds for the duration of the exercise. The vertical lines indicate the time point that the power on the bike was changed and the numbers on top of the graph represent the power level (Watts) at which the subject was cycling. **c** Mean absolute percent error for various wearable devices during total sleep time. The numbers denoted next to each bar represent the mean absolute percentage error values which were used to calculate the absolute difference between each monitor and the sleep diary values. Figures were reproduced with permission from Stahl et al.^102^ **a**, Farzam et al.^117^ **b**, and Lee et al.^131^ **c**

Despite demonstrated use of wearable sensors for HR detection, the assessment of their accuracy (defined as the statistical difference between actual and reported data) is still limited. A study by Wang et al. highlighted these limitations in a number of wrist-worn heart monitors, such as Polar H7, Apple Watch 3, Mio Fuse, Fitbit Charge HR, and Basis Peak.^100^ Heart rate readings were compared to a gold-standard ECG and it was found that the Polar H7 device had the highest concordance correlation coefficient of 0.99 followed by the Apple Watch (0.91), Mio Fuse (0.91), Fitbit Charge HR (0.84), and Basis Peak (0.83). The study found that none of the wrist-worn devices achieved the accuracy of a chest-strap-based monitor. Additionally, these devices were most accurate when measuring resting HR and became less accurate with increasing exercise. Gillinov et al. assessed the accuracy of four optically based HR wrist-monitors (Apple Watch, Fitbit Blaze, Garmin Forerunner 235, and TomTom Spark Cardio), one on each wrist, and one forearm monitor (Scosche Rhythm+) compared to an ECG chest strap monitor (Polar H7) during various types of aerobic exercise.^101^ Fifty healthy adult volunteers performed exercise protocols on a treadmill, a stationary bicycle, and an elliptical trainer (arm movement). HR was recorded at rest, light, moderate, and vigorous intensity for each exercise. Agreement between the HR measurements from the wearable sensor and that of ECG was assessed using Lin’s concordance correlation coefficient (*r*_c_). The chest strap monitor (Polar H7) had the best agreement with ECG (*r*_c_ = 0.996) across all exercises followed by the Apple Watch (*r*_c_ = 0.92), the TomTom Spark (*r*_c_ = 0.83), and the Garmin Forerunner (*r*_c_ = 0.81), Scosche Rhythm+ (*r*_c_ = 0.75), and Fitbit Blaze (*r*_c_ = 0.67). All devices performed well (*r*_c_ = 0.88–0.93) on the treadmill except the Fitbit Blaze (*r*_c_ = 0.76). During cycling, only the Garmin, Apple Watch, and Scosche Rhythm+ had acceptable agreement (*r*_c_ > 0.80). On the elliptical trainer without arm levers, only the Apple Watch was accurate (*r*_c_ = 0.94). None of the devices were accurate during elliptical trainer use with arm levers (all *r*_c_ < 0.80). The study found that the accuracy of wearable, optically based HR monitors varied with exercise type and was greatest on the treadmill and lowest on the elliptical trainer. The team concluded that electrode-containing chest monitors should be used only when accurate HR measurement is needed. In another similar study, Stahl et al. evaluated the accuracy of the Scosche Rhythm (SR), Mio Alpha (MA), Fitbit Charge HR (FH), Basis Peak (BP), Microsoft Band (MB), and TomTom Runner Cardio compared to the Polar RS400c HR chest strap among 50 healthy volunteers (Fig. 4a).^102^ The study protocol entailed having the volunteers on a treadmill for 30 min walking at various velocities for 5 min each (3.2, 4.8, 6.4, 8.0, and 9.6 km/h). Interestingly, the study by Stahl et al. showed that wearable activity trackers provided an accurate measure of HR during non-stationary activities. The mean absolute percentage error values were: 3.3%, 3.6%, 4.0%, 4.6%, 4.8%, and 6.2% for the TT, BP, RH, MA, MB, and FH wearable wrist-devices, respectively. The Pearson product–moment correlation coefficient (*r*) was calculated: *r* = 0.959 (TT), *r* = 0.956 (MB), *r* = 0.954 (BP), *r* = 0.933 (FH), *r* = 0.930 (RH), and *r* = 0.929 (MA). Results from a 95% equivalency test showed that monitors were found to be equivalent to those of the criterion HR (±10% equivalence zone: 98.15–119.96). The authors of this review hypothesize that these deviations can be attributed to various factors, such as the modality of the PPG signal compared to that produced from an ECG (a key difference between wrist-monitors versus epidermal patches), the difficulties associated with peripheral wrist location, and the noisy interface of the skin.^103,104^ Further clinical validation and standardization of clinical protocols may be necessary to homogenize results among clinical trials to accurately improve patient outcomes via the use of such technology.

There has been a growing interest to develop epidermal electronics to monitor HR, leveraging advancements in flexible materials and health analytics to mitigate accuracy-related issues posed by wrist-worn devices. Epidermal patches such as the BioStamp MD™, Healthpatch MD, Vital Scout, and Zio XT Patch, have emerged as promising options for monitoring the HR of athletes. Kabir et al. utilized the MC10 Biostamp to develop an optimal configuration of the sensors, which would provide the best agreement with the Frank orthogonal ECGs for long-term monitoring of a vectorcardiogram (VCG).^105^ A VCG represents the movement of the heart vector in three orthogonal dimensions and provides information complementary to that of a 12-lead ECG.^106^ Analysis of VCGs has been demonstrated to help define abnormal electrophysiological substrate in patients with life-threatening ventricular arrhythmias and sudden cardiac death (SCD).^106^ The study by Kabir et al. evaluated parameters, such as the QRS-T angle, spatial QRS, and T-vector characteristics, and other global electrical heterogeneity parameters in 50 subjects.^105^ Each subject underwent 10 s of orthogonal ECG followed by 3–5 min of ECGs using the Biostamp patches and MAC 5500 ECG system at five locations on the torso while at rest in a sitting position. Results confirmed that the biostamp patches could be used for the long-term monitoring of the VCG parameters previously described. Utilizing devices like the Biostamp demonstrates the utility of epidermal ECG patches to monitor those at risk of arrhythmias in a non-intrusive and continuous manner. Translation of such technology to monitor athletes suffering from cardiac conditions, such as hypertrophic cardiomyopathy or atrial fibrillation present next steps to further enhance the value of wearable sensors for sports. The American College of Cardiology (ACC) recently established the Sports and Exercise Council.^107^ An important objective of this council is to define the essential skills necessary to practice effective sports cardiology to track competitive athletes and highly active people (CAHAP) who may be most at risk for adverse cardiovascular outcomes during intense physical activity.^107^

Building upon the work by Kabir et al. clinical adaptation of devices like that of the Vital Scout patch by VivaLNK in sports such as rowing could diversify the use case of such technology to proactively monitor the health of athletes in a real-time manner to gain insight regarding their heart rate, respiration rate, stress levels (as a function of heart rate variability, HRV, as discussed later in this review), sleep, and activity.^108^ In a recent study, Lee et al. fabricated a self-adhesive ECG patch that conformally laminated onto the wrinkles of the skin, maintained robust contact, and self-adhered onto the epidermis.^109^ The epidermal device recorded various biosignals from an ECG, EMG, and electrooculogram (EOG) while avoiding skin irritation. The team developed a multi-material dry adhesive utilizing polydimethylsiloxane (PDMS) and carbon nanotubes (CNTs), leveraging the biocompatibility and excellent mechanical properties of the polymer and electrical conductivity of the CNTs. The device showed promise for monitoring ECG signals long-term. In another study, researchers developed a multifunctional epidermal device capable of measuring biosignals from both ECG, EMG, and temperature.^110^ The sensor contacted the skin directly via an elastomeric stamp which was transfer printed onto the skin via the application of an acrylate/silicone spray-on-bandage. The study demonstrated that the application of advanced materials coupled with integration methodologies resulted in a viable multimodal epidermal sensor for monitoring responses through and on the skin. In another study, Hu et al. developed a conductive elastomeric electrode devoid of conductive pastes for the measurement of an ECG signal.^111^ PDMS was mixed with other biocompatible conductive nanoparticles to improve the electrical conductivity of the substrate. A micro-replica mold casting for the micro-structures was applied to reduce the micro-structural deformations along the direction of signal transmission to maintain the corresponding electrical impedance under the physical stretch by the movement of the human body. The gel-less electrodes provided a more convenient and stable bio-potential measurement platform when tested on a healthy human subject undergoing walking and running tests. Further work translating such technologies on athletes during real-time practices remains the next step before the efficacy of such devices is deemed ready for everyday use. Specifically, there remains a need to evaluate the build-up of eccrine sweat underneath the substrate of such devices to study skin health and sensor adhesion over a prolonged workout.

Another emerging area to monitor HR for athletes is the incorporation of sensors into textiles to form “smart garments”. Researchers fabricated a wearable ECG monitoring garment that utilized electrodes from carbon-derived paste.^112^ The paste was applied to the skin and dried for 5 min resulting in a flexible and detachable electrode. The electrodes were connected to an ECG affixed to the garment and used to measure ECG signals during walking and running. Despite the promising research, we assessed that issues such as reliability of electrode connections (as a function of player movement), adhesive robustness and longevity (due to eccrine sweat generated during workouts), and the effects of impact on sensor reliability need to be considered during preliminary studies to test if the device is capable of monitoring real-time performance in sports. On the commercial front, Kymira, a smart textile company, launched an early prototype, currently in final development, of its cardiac monitoring t-shirt designed to lower the risk of heart attacks in athletes.^113^ The shirt wirelessly transmits the athlete’s heart rhythm to a mobile phone via Bluetooth where it can identify an unusual heart rhythm that could lead to sudden cardiac arrest. Printed electrodes on the shirt’s fabric feed into a processing unit which transmits and rectifies the ECG data. The textiles in the shirt regulate body temperature to improve athletic performance. Furthermore, minerals embedded in the fabric capture energy produced by the body during exercise and re-emit that back as infrared (IR) energy into muscle. This has been shown to increase circulation, increase tissue oxygen levels up to 20%, and provide pain relief to reduce muscle soreness.

The value in HR training is in the use of zones, which are all based off an HR value that is relative to the maximum HR. Despite its potential value for sports, there remains multiple issues with HR monitoring.^114^ Firstly, maximum HR is often calculated using the formula “220 minus the age of the person”, or a slight variation to that. The physical fitness, body composition, or other individual variances which could affect this maximum HR value are not considered. Secondly, HR is dependent on multiple external factors, such as caffeine intake or sleep. Thus, HR may be unreliable unless it is measured under extremely controlled environments. Lastly, HR is a systemic measurement. It provides information on how the heart is adjusting to activity; however, it fails to provide specific information about how the working part of the body is responding to exertion. As such, measurement of an HR value currently provides little to no use when athletic trainers want to quantify the workout of the athlete to determine the ACWR and to predict the incidence of soft-tissue injuries during the rigors of training camp or live performance. As reviewed next, measurement of muscle oxygen saturation (SmO_2_) levels has been shown to circumvent such hurdles.

### Muscle oxygen saturation

Physiological quantification of how muscles respond to physical exercise is gaining interest in elite-level athletes to improve their overall performance. In the past, athletes have relied upon measurements, such as blood lactate concentration, HR, or maximum oxygen uptake (VO_2max_) to assess the intensity at which they should be exerting themselves.^115,116^ While quantification of these parameters has helped craft athlete-specific workout regimens to improve performance, these measurements are indicative of systemic changes in the body, with no detailed information about the working muscle groups.^117^

Muscle oxygen saturation, which refers to the amount of oxygen in the blood of muscles, is a measurement that has emerged as a useful parameter to help athletes optimize their performance. The technology behind SmO_2_ monitors was developed several decades ago; however, it remains an emerging area for wearable device fabrication in scientific literature today. Commercial wearable, fiberless devices include the Humon Hex, Moxy Monitor (Fortiori), and Portamon (Artinis Medical System).^117^ These devices work by non-invasively measuring the amount of oxygenated and deoxygenated blood in the muscles using light waves.^117^ The Moxy Monitor and Portamon devices can be manually strapped on to any muscle group and have been used during a variety of activities including cycling, running, and strength training.^117^ The Humon Hex is 6.0 × 5.7 × 1.4 cm in size and is placed over the quadriceps using a Velcro strap that hooks through the device^117^ (Table 4). The device communicates with a smartphone via Bluetooth and a custom app that displays the workout progress in real-time. Muscle oxygen monitors (such as the ones mentioned) use optical techniques to measure the oxygenated hemoglobin concentration (HbO_2_) and deoxygenated hemoglobin concentration (Hb) in the muscle during exercise.^117^ These devices are able to do this by shining near-infrared light (NIR, 0.7–1.4 µm wavelength) into the muscle and by detecting reflected light. Hemoglobin concentrations can be quantified by measuring the amount of light that is absorbed.^118,119^ The parameter that is typically reported to the athlete is called muscle oxygen saturation (SmO_2_), which is the ratio of HbO_2_ to total hemoglobin concentration (HbT), where HbT is the sum of HbO_2_ and Hb.^114,117^ As the muscles exert more energy (work harder), more oxygen is used and the SmO_2_ level decreases.^120,121^ Therefore, SmO_2_ provides athletes with a localized measurement for how muscles are performing during an activity. Some of the benefits from using SmO_2_ are in (1) measuring localized muscle performance, (2) determining whether the working muscles are being exerted beyond their limits to inform the athlete that their muscles are running low on oxygen and they (athlete) cannot sustain the current activity, and (3) evaluating muscle recovery. SmO_2_ can show the rate at which the oxygen is delivered back into the muscles and when the muscles are ready to perform again.^122^ There are two primary factors that influence SmO_2_ measurement throughout exercise: oxygen delivery and oxygen consumption.^120,121^ As the muscles exert more effort, they demand more oxygen thus increasing blood supply. This increase is accomplished primarily by an increase in HR. However, there is a level when this increase in blood supply can no longer match the oxygen demand within the muscle, which results in a decrease in SmO_2_ levels. When the athlete slows down during the recovery phase of an interval set, the SmO_2_ increases due to the lower oxygen requirement in the muscles in addition to the high blood supply still being present.^117^

A key question left unanswered in the field is: Can the integration and translation of SmO_2_ levels measured from wearable sensors into internal workload models (HR or sRPE) better monitor athletic performance and predict soft-tissue injury in elite-level athletes? As previously mentioned, the sRPE is based on the intensity of the session on a 1–10 ‘rating’. However, it does not consider key physiological parameters which play a crucial role in the performance of the athlete. We hypothesize that the stratification of physiological parameters (e.g. SmO_2_ levels) into a scale analogous to that of current sRPE ratings to calculate player loads would enable intrinsic workload measurements to be based on physiological parameters which affect workout intensity and performance rather than a scale lacking formal clinical guidelines and variability among athletes. We predict that development of such models from sensor data could serve as the next-gold standard to accurately and efficiently assess human performance.

Clinical validation of current devices against benchtop technology is needed to enable this translation. Farzam et al. compared the SmO_2_ levels by the Humon Hex (beta device) against a benchtop fiber-based frequency-domain NIR (FDNIRS) system (MedaOx, ISS) on the rectus femoris muscle among 14 male and 3 female athletic subjects on a cycle ergometer.^117^ The goal of the study was to examine the accuracy of the Humon Hex to understand potential limitations in measuring SmO_2_ levels. The authors studied the real-time feedback from the Humon device and reported variations between the optically derived threshold and blood lactate threshold during an incremental cycling test. The rectus femoris was selected to maximize fiber movement, since this was the area of the leg where the fibers remained the most stable. In addition to the body mass index (BMI) calculated for each subject, the subcutaneous adipose tissue thickness (SCATT) on the rectus femoris of the right quadricep was measured using a skinfold caliper before the start of the cycling test. Blood lactate levels were calculated based on a combination of HbO, HbR, HbT, and SmO_2_ levels and were displayed among corresponding exercise zones (green, orange, red, and blue). The green zone indicated a steady state. The orange zone indicated the athlete is approaching their limit. The red zone indicated the athlete has hit or exceeded their limit. The blue zone indicated that the athlete is in recovery phase. Overall, the study validated the performance of the Humon Hex wearable device against the MedaOx benchtop system (Fig. 4b). The wearable device provided similar results to more expensive FDNIRS technology. The main limitations to all continuous wave (CW) and FDNIRS systems is the reduced sensitivity to muscles in the presence of subcutaneous adipose tissue.^117^ Focusing on athletes who tend to have thin adipose layers provides a larger drop in SmO_2_ levels than what can be achieved of those with thicker adipose layers.^117^ Monitoring the sleep quality of the athlete is instrumental for the athlete to maintain a healthy HR and SmO_2_ range necessary to maximize performance.

### Sleep quality detection

Sleep quality and duration is an important measure of health and is known to directly affect the performance and recovery of an athlete. Wearable devices have been developed to evaluate sleep quality and have focused on monitoring body movement patterns as a measure of sleep restfulness. Examples of wearable devices currently in the market which monitor and track sleep quality are the Fitbit sensors, Jawbone UP, Misfit Shine, Komodo AIO Smart Sleeve, Polar watches, and WHOOP band (Table 5). The WHOOP band is the first wrist-based device to proactively prescribe to the user the hours of sleep needed to ensure a full recovery. The device measures physiological markers (e.g. resting HR, HRV) to indicate strain, optimize recovery, and maximize performance on a daily basis.^123^ Based on this data, the algorithmic platform determines the physical exertion during workouts over the course of a day and utilizes this data to estimate the number of hours of sleep required for a full recovery.^123^ A recent study, funded by WHOOP, utilizing the WHOOP band, compared changes in and relationships between resting HR, HRV, and sleep characteristics in 10 NCAA Division 1 collegiate female cross-country athletes over a 12-week season.^124^ Resting HR at the end of the season showed a meaningful increase compared to the beginning of the season. Higher resting HR and lower HRV were associated with an increase in percentage of time spent in a slow wave sleep. The data suggested that when the physiological restorative demand was higher, the percentage of time in slow wave sleep was increased to ensure recovery. The study demonstrated that monitoring sleep using devices like the WHOOP band enabled the implementation of sleep hygiene strategies to promote adequate slow wave sleep when the body needed physiological restoration. Randomized controlled studies comparing the WHOOP band to other devices utilizing larger sample sizes among athletes is greatly needed to validate the efficacy of such technology for athletes.

Various studies have shown that a lack of sleep lowered athletic performance, worsened lung function, decreased the time to fatigue, increased injury risk, and increased lactic acid production thereby increasing the likelihood of post-workout muscle fatigue and soreness.^125–130^ A recently published study assessed the accuracy of commercially available sensors in 78 adults with a mean age of 27.6 ± 11 years by estimating sleep measurement with a sleep diary (SD) for three nights.^131^ Results showed that the greatest equivalence with the SD for total sleep time were the Jawbone UP3 and fitbit charge heart rate with effect sizes of 0.09 and 0.23, respectively (Fig. 4c).^131^ Other tested wearables such as SenseWear Armband, Garmin Vivosmart, and Jawbone UP3 produced the greatest effect sizes of 0.09, 0.16, and 0.07 respectively.^131^ Rosenberger et al. assessed the accuracy of nine wearable devices (Actigraph GT3X+, activPAL, Fitbit One, GENEactiv, Jawbone Up, LUMOback, Nike Fuelband, Omron pedometer, and Z-Machine) over a 24-h period in their ability to accurately track sleep.^132^ The sedentary behavior (SED), light intensity physical activity (LPA), and moderate-to-vigorous physical activity (MVPA) were measured. The Z-Machine utilized three electrodes applied to the head/neck to measure sleep. The other devices were worn on the wrist and relied on an accelerometer-based measurement algorithm to estimate total sleep time. LUMOback and activPAL did not have specific sleep measurement because sedentary time and sleep were recorded based on posture and were excluded from sleep measurements. The Fitbit device was moved from the trunk to the wrist and placed over the forehead for sleep measurement. The subject then pressed and held a button on the device to enable sleep mode. Similar procedures were used for the Jawbone, GT3X+, and GENEactiv devices. Comparisons (to standards) were derived for total sleep time (Z-machine), time spent in SED (activPAL), LPA (GT3x+), MVPA (GT3x+), and steps (Omron). Error rates ranged from 8.1–16.9% for sleep, 9.5–65.8% for SED, 19.7–28.0% for LPA, 51.8–92% for MVPA, and 14.1–29.9% for steps. The GT3X+ device had the closest measurement for sleep, LUMOback for sedentary behavior, GENEactiv for LPA, Fitbit for MVPA and GT3X+ for steps. The study concluded that no device accurately captured activity data across an entire day. Polysomnography (PSG) remains the gold standard for monitoring sleep and should be utilized in clinical studies to quantify the efficacy of a wearable device to track sleep.^133^ PSG involves recording multiple physiologic variables, including EEG, ECG, EMG, and electro-oculogram (EOG), which is then scored by human examiners based on standardized criteria.^134^ While PSG recordings provide an accurate measurement of sleep quality, their high cost make it impractical to implement within a long-term sleep monitoring system. Furthermore, attaching numerous sensors to an individual’s body is considered intrusive, and may in turn disturb sleep. It has been hypothesized that wearable sensors could bridge this gap. Mantua et al. assessed the reliability of wrist-worn wearable devices, such as the Basis Health Tracker, Misfit Shine, Fitbit Flex, Withings Pulse O2, and a research-based actigraph, Actiwatch Spectrum against PSG.^135^ A Wilcoxon Signed Rank test was used to assess differences between devices relative to PSG and to correlate the strength of the data. Data loss was greatest for the Fitbit and Misfit devices. For all the devices, the authors found a strong correlation of total sleep time with PSG; however, sleep efficiency differed from PSG for the Withings, Misfit, Fitbit, and Basis devices. Data from the Actiwatch did not differ from that of PSG. A weak correlation in sleep efficiency (time asleep/time in bed) was noted from Actiwatch correlated with PSG. Light sleep time differed from PSG (nREM1 + nREM2) for all devices. Measures of deep sleep time did not differ from PSG (SWS + REM) for the Basis device. While total sleep time, and in some cases, sleep efficiency, can be monitored via wrist-worn, devices, the reliability of these sensors remains low. Furthermore, the authors concluded that these devices did not yet yield sufficient information for accurate sleep staging, even on a superficial level (e.g., light vs. deep). The authors concluded that PSG should remain the mainstay when monitoring the sleep of individuals. Further studies of devices against PSG are necessary to test the clinical relevancy of this technology for elite-level athletes.

## Next steps: wearable sensors assist in the return to play for athletes

Sports medical personnel are faced with return-to-play (RTP) decisions for every athlete who want to return to activity at the highest level.^136^ The myriad of factors related to history, physical examination, testing, workload and intensity, and baseline characteristics of the athlete can make the RTP decision-making process complex and challenging.^136^ The RTP decision-making process was authored in a three-step protocol referred to as the Strategic Assessment of Risk and Risk Tolerance (StARRT).^136,137^ Step one outlines the medical factors associated with the injury to determine the level of injury severity with the RTP.^136^ Step two focuses on the player or sport factors that may mitigate or augment the risk of injury or reinjury.^136^ Step three is focused on the factors associated with whether the final ascertained risk is worth taking within the confines of the needs of the coach, team, athlete and medical service provider.^136^ While inclusion of steps one and three are key to the final decision, our discussion in this review was focused on step two, specifically on the initial evaluation and monitoring of the athlete to assess their performance and risk of initial or reoccurring injury.

Our discussion in this review was centered around the ability of the musculoskeletal, cardiopulmonary, and psychological systems of the athlete to be assessed and quantified in a non-invasive and unobtrusive manner to restore sports-specific skills and function. We believe a key aspect that has been excluded from the RTP decision is related to quantitatively measuring the amount of training the athlete has completed over the time of the recuperation or during high-acuity training periods to ultimately enable the athlete to be mentally and physically prepared for the physical and mental demands of the game (Table 6). A central theme which permeated throughout this review was that the use of wearable sensors can enable medical personnel and athletic trainers to monitor the biomechanical and physiological status of the athlete to mitigate or minimize the onset of injuries and assess athlete performance in a real-time manner. Risk of athlete participation is dependent on the interaction between tissue health (biomechanical, physiological, or mental stress the tissue can absorb) and tissue stresses (biomechanical, physiological).^136^ This risk is then compared with the clinician’s and/or athlete’s risk tolerance, which is a function of numerous factors related to the overall health of the athlete.^136^ After all factors considered, if the risk assessment is less than the risk tolerance, the decision should be to RTP.^136^ Conversely, if the risk assessment is greater than the risk tolerance, the decision should not be to RTP.^136^

**Table 6:** Summary of methods utilized or emerging to quantify athlete training load to monitor recovery and performance

Method	Used today in sports	Wearables utilized	Metrics	Advantages/disadvantages
Questionnaire	Yes	No	Verbal or written form	*Advantage*: Easy to conduct
				*Disadvantage*: Highly variable; often inaccurate
Session-rate of perceived exertion	Yes	No	Scale from 1 to 9 detailing intensity of workout. Scale used in conjunction with workout duration to determine load	*Advantage*: Easy to assess
				*Disadvantage*: Highly variable; often inaccurate
Blood lactate	Yes (emerging)	No	Concentration	*Advantage*: Used to predict anaerobic threshold (kicks in when exercise is increased and the aerobic system can no longer keep up with the body’s energy system
				*Disadvantage*: Cost, inefficient, time-varying process
Tri-axial accelerometers and GPS	Yes	Yes: Catapult, Zebra	Acceleration, location, and velocity used to compute PlayerLoad (arbitrary unit) to derive ACWR	*Advantage*: Easy to utilize
				*Disadvantage*: Variability in sensor technology could lead to inaccuracy. Need to develop algorithms to filter noise (e.g. player moving on the sideline compared to on-field performance)
Heart rate	No	Yes: Apple Watch, Fitbit, Polar	Time in HR zones, HRV	*Advantage*: Easy to collect large data sets for robust analysis
				*Disadvantage*: Variability in sensor technology could lead to inaccuracy. Sensor location attributed to deviations.
Muscle oxygen saturation	No	Yes: Humon Hex	SmO_2_ levels stratified into workout zones	*Advantage*: Easy to collect large data sets
				*Disadvantage*: Need for validation of models
Biochemical concentration^5–8^	No	No devices used to monitor training load and recovery directly. Indirect measures include monitoring hydration levels and sweat rate	Concentration	*Advantage*: Insight into the biochemistry of the athlete to predict hypohydration, hyponatremia, and fatigue.
				*Disadvantage*: Technology still developing. Need to develop predictive analytics based on the biochemical profile of the athlete

In summary, this paper discussed the utility of wearable sensors to measure biomechanical and physiological parameters affecting athlete performance. Specifically, the first section on physical performance and safety included sensors which measure position and motion, impact, and biomechanical forces. The second section pertaining to the physiological status of the athlete included sensors which measure heart rate, muscle oxygen saturation, and sleep quality. In each section, we provided examples to discuss how such technology has been utilized or could be adapted by the sports community to enable athletes to perform better, recover faster, and stay safer.
